# Health Care and Health Information Access by Parents With Young Children in Regional Queensland

**DOI:** 10.1111/ajr.70060

**Published:** 2025-05-30

**Authors:** Catherine McCosker, Gavin Beccaria, Lisa Beccaria, Tanya Machin

**Affiliations:** ^1^ School of Nursing and Wellbeing University of Southern Queensland Toowoomba Queensland Australia; ^2^ School of Psychology and Wellbeing University of Southern Queensland Toowoomba Queensland Australia

**Keywords:** children, general practitioners, health information, internet, parents, rural

## Abstract

**Objective:**

The effects of childhood health, education and experiences can have long‐term impacts on adult health and wellbeing. Access to health services and information can be complex especially in regional and rural areas of Australia. This research aimed to: (1) investigate how and where parents living in regional and rural Australia with young children search for health information and (2) explore how parents decide what is appropriate health information to enable them to meet the health needs of their families.

**Setting:**

Regional and rural areas of Southern Queensland.

**Participants:**

Parents with a child under the age of 5 years.

**Design:**

A convergent mixed methods design was utilised. Parents participated in an online survey and were invited to in‐depth semi‐structured telephone interviews about their health information search methods. Inductive content analysis was applied to the transcripts.

**Results:**

The 11 interviewees searched for health information when their child was unwell, using the internet, family and friends and GPs and medical services. Websites were used for health information, whereas social media sites provided support and connection. The internet helped determine when to seek medical advice, and a preference was shown for Australian, hospital and government websites and websites recommended by GPs.

**Conclusion:**

The results may inform the development of targeted hospital and government websites to ensure all parents have easy access to evidence‐based children's health information. GPs may also play a role in discussing internet‐sourced health information with parents.

**Summary**

- What is already known
  ○ Parents commence looking for information by using the first listed options appearing via the internet search engines.
  ○ Parents use hospital and government web sites as a source of credible information.
  ○ Presentation of website health information influences parent's perceptions of the quality of the information.
- What this study adds
  ○ Parents look for information related to illness, rather than wellness.
  ○ Parents use web pages for health information and social media sites for support and connection.
  ○ Parents use information retrieved from the internet to determine the urgency of GP appointment.

Health care access is a dynamic and often complex concept, yet it is central to maintaining health and wellbeing [1]. The Patient‐Centred Access to Health Care conceptual framework proposed by Levesque et al. (2013) (see Figure 1) suggests health care users must have the ability to perceive the need for health care and to seek, reach, pay for and engage with health services to enjoy better health outcomes. Equally important to health outcomes are health care services that are approachable, acceptable, available, affordable and appropriate [1]. Accessing appropriate health care and health information for a young child is not always an easy task for parents. The importance of early childhood is well recognised as the long‐term health and welfare implications for the child persist into adulthood and are well documented in the literature [2, 3] Health services and health information related to young children can be difficult for parents to navigate as it is often complex and complicated.

**FIGURE 1:**
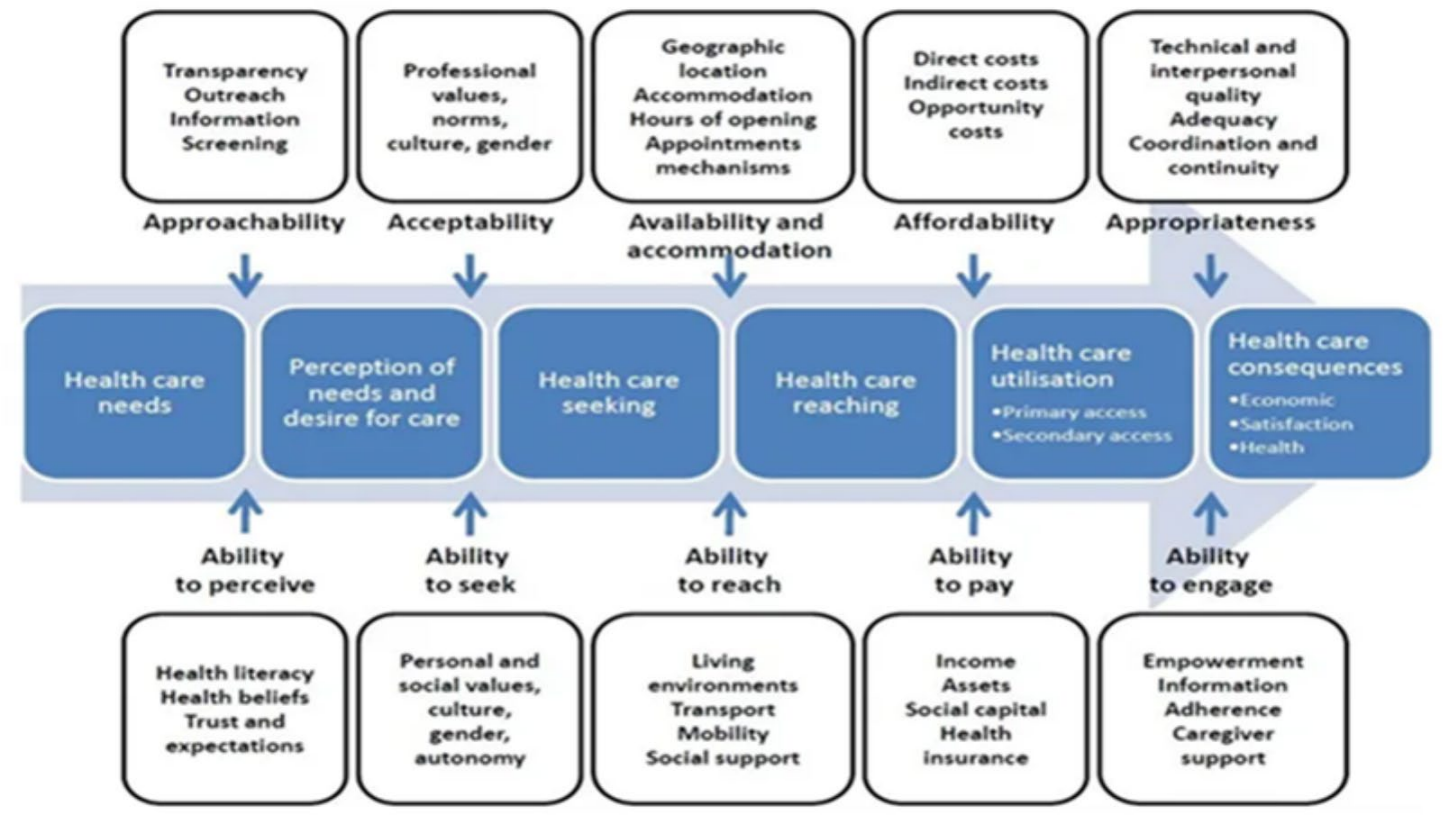
Conceptual framework of Patient‐Centred Access to Health Care. Levesque et al. [1]

Traditionally, Australian families have sought the advice of their local doctor when concerned about their child's health [4]. However, due to the widespread shortage of General Practitioners (GPs) in Australia, notably in regional and rural areas, access to primary health care services is becoming increasingly difficult [5]. Families must seek health care and health information from alternate sources. For example, in regional Queensland, low socioeconomic families have been noted to access acute care services more frequently [6]. There is some evidence in the literature indicating internet access can improve an individual's access to health care and thereby reduce health inequalities [7]. Conversely, concern has been expressed that poorer access to internet services and inadequate internet skills could create a ‘digital divide’ contributing to further inequities [8, 9, 10].

Health literacy has been identified as an important component of the ability to perceive health care needs [1]. Parents with low health literacy tend to overestimate the severity of their child's illness and to seek care sooner [11]. They also tend to utilise the emergency department (ED) more frequently for their child [12, 13], and to present more frequently with non‐urgent conditions for their child [14]. Children, whose parents have low health literacy, are less likely to experience preventative health care, have less home safety awareness and are more likely to miss medication doses and be exposed to environmental cigarette smoke [15, 16]. The rising use of technologies and online health services, particularly following the COVID‐19 pandemic, has added another dimension in the search for health information. Digital health literacy suggests the need for extra skills within the context of health literacy. Specifically, inadequate computer literacy skills have been identified as a barrier to effective online searching [17], and despite the widespread use of more accessible technologies such as smartphones and tablets [18], poor digital health literacies persist [9]. People with low literacy levels have reported greater difficulty generating search terms and have a greater reluctance to use hyperlinks [19]. These individuals tend not to question the quality of online information [20], and have more difficulty evaluating online health information [21] Parents with low health literacy, who sought non‐urgent care for their children at the hospital ED, were less likely to use the internet for health information [22].

To retrieve information rapidly and to personalise information, internet search engines use algorithms or sets of rules [23]. Algorithms make use of personal data such as search history and contextual information, such as search language and location, to identify, prioritise and personalise the information presented [23]. Therefore, these algorithms control the flow of information received by a searcher by presenting a small selection of information and making recommendations of further information [24]. This has implications for parents in terms of finding credible, reliable and consistent information that is appropriate for their child's needs. There is limited discussion in the literature about how and where regional and rural families access health care and health information for their young children, and what difficulties they encounter while trying to care for the health of their children. Therefore, this study aims to address this gap by examining how parents and/or caregivers search for health information and how they decide what is appropriate information to meet the health needs of their children.

## 1 Method

A convergent mixed methodological design was utilised to address the research questions. The project received ethics approval from the host university. The target participants were parents with children under the age of 5 years, and who resided in regional and rural southern Queensland Australia. Advertisements inviting parents to participate in a short online survey about where they sought children's health information were distributed through daycare centres, playgroups and kindergartens, via poster, flyers and were also incorporated into electronic newsletters. The recruitment of survey participants proved to be challenging, necessitating an expansion of the recruitment area to the broader Southwest Queensland region. The use of snowballing techniques, such as social networking and promotion of the survey through family support programs, and advertising in public places such as local supermarkets and library community notice boards were also employed. Participants accessed the survey through either a QR code or a hyperlink. The survey, which was designed and tested by the authors, contained a total of 16 questions and took < 10 min to complete. The initial questions identified if the participant met the inclusion criteria, that is, having a child under the age of 5 years, residing in the local rural and regional areas, holding an Australian Medicare Card and being an English language speaker. Further questions targeted demographic information, such as education and employment, and identified what health sources the parents had accessed in relation to their child. Health literacy was screened using the Single Item Literacy Screener ‘How often do you need to have someone help you when you read instructions, pamphlets or other written material from your doctor or pharmacy?’ [25]. All survey participants who fully completed the survey, were entered into a prize draw for a $40 grocery gift card, with the winner being randomly selected and notified via email.

At the completion of the survey, respondents were invited to participate in an in‐depth interview about how and where they sought health information for their child. Those participants who consented to further contact were given an information sheet outlining the purpose and processes of the interviews. Informed consent was obtained from the participants prior to the interviews. All interviews were conducted via telephone by CM at a prearranged time. The interviews were recorded using a password protected portable digital voice recorder, with notes being taken by the interviewer throughout. An hour was allocated per interview. The interviews were transcribed manually and deidentified. Individual transcripts were available to the participants for comment on request. However, no participant requested a transcript of their interview. The interviews were semi‐structured, with a core of open‐ended questions. Examples of these core questions included but were not limited to: (1) where do you go for health information about your child? (2) when do you use the internet to look for health information about your child? and (3) describe the features you look for when choosing an internet source of information? All interviewees received a $50 grocery gift card on completion of the interview.

The interviewer (CM) was a Registered Nurse employed in Child Health with an interest in promoting healthy outcomes for children. This study formed part of a PhD project. There were no professional or personal relationships between the participants and the interviewer, as per the Ethics approval.

**FIGURE 2:**
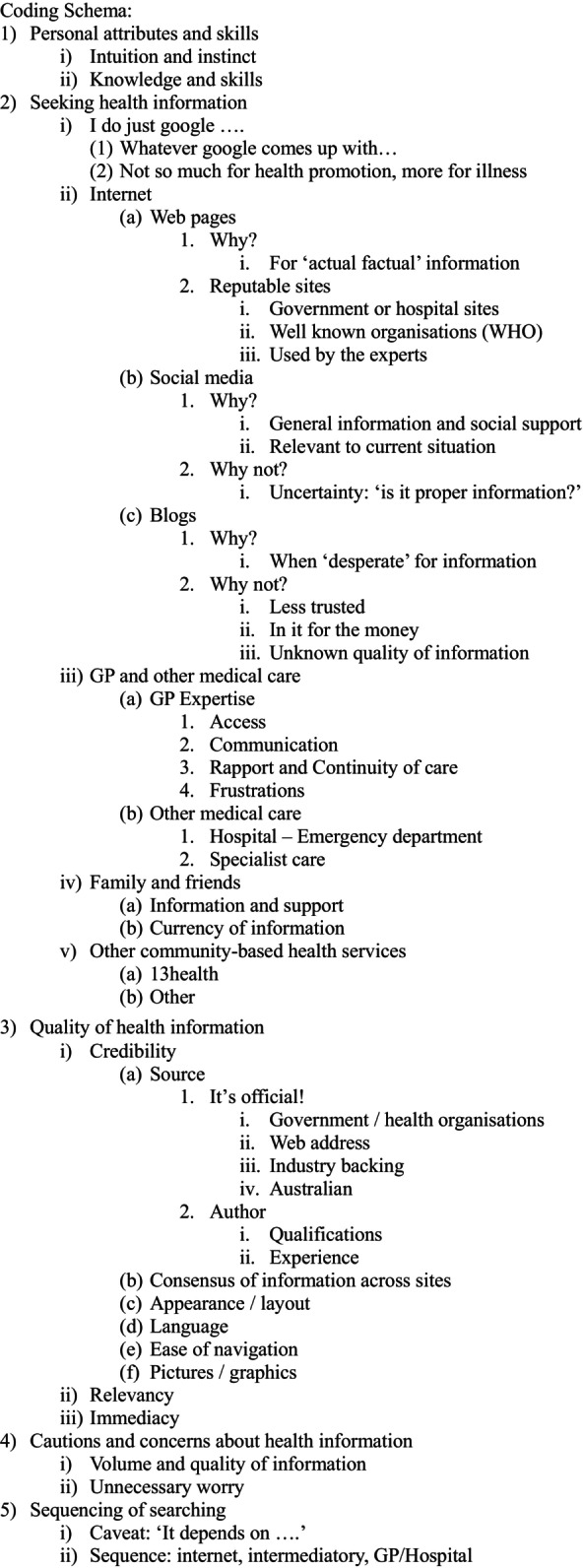
Refined coding schema.

Inductive Content Analysis was applied to the transcribed interviews. This method of qualitative analysis was chosen it as was best suited to the practical, health orientation of the research [26]. Iteration initially identified the ‘big picture meaning units’ and was repeated to identify ‘categories’ and then ‘fine grained codes’. The final iteration led to the development of the ‘refined coding schema’ [26]. The iteration and coding process was reviewed by the research team. The resultant coding schema (Figure 2) addresses the research questions in a clear structured format.

## 2 Findings

### 2.1 Survey

Data from 33 online surveys were received; however, five surveys were incomplete and were therefore excluded from further analysis. All 28 completed surveys met the inclusion criteria. As displayed in Table 1, only 18% (*n* = 5) of respondents held an Australian Concession/Health Care Card (AC/HCC), and less than 1% (*n* = 2) identified as First Nations people. The responses indicated 71% (*n* = 20) had attained post high school qualifications through trade or university, 89% (*n* = 25) were either in full‐time, part‐time or casual work, and 82% (*n* = 24) of respondents' partners were also in paid employment. The GP was accessed for their child by 89% (*n* = 25), and 71% (*n* = 20) had utilised the ED. Only 32% (*n* = 9) had used 13 Health^1^ or the Poisons Information service, and 46% (*n* = 13) had used Child Health services. Most respondents, 85% (*n* = 24), had used the internet to look for health information for their child and did not need help reading information from the doctor or pharmacist. The survey respondents differed from the wider community particularly in the areas of education, holding an AC/HCC and identification as First Nations peoples, as shown in Table 2.

**TABLE 1:** Demographic information of survey respondents.

	Number	Percentage
Australian Medicare card		
No	0	0
Yes	28	100
Australian Concession Card/Health Care card (AC/HCC)		
No	23	82
Yes	5	18
Ethnicity		
First nations	2	< 1
Not first nations	25	89
Non‐English speaking background	1	< 1
Education		
Did not finish high school	3	10
Completed high school	5	17
Trade or similar qualification	7	25
University qualification	13	46
Paid employment		
No	3	10
Regular full‐time	9	32
Regular part‐time	12	42
Casual	4	14
Partner employment		
Not applicable	3	10
No	1	< 1
Regular full‐time	23	82
Regular part‐time	0	0
Casual	1	< 1
Accessed emergency department in previous year		
No	8	28
Yes	20	71
Accessed GP/medical practitioner in previous year		
No	3	10
Yes	25	89
Accessed 13Health or Poisons Information in previous year		
No	19	67
Yes	9	32
Accessed child health services in previous year		
No	15	53
Yes	13	46
Accessed internet for health information in previous year		
No	4	14
Yes	24	85
Single question Health Literacy Screen (SILS)		
Never	24	85
Rarely	4	14
Sometimes	0	0
Often	0	0
Always	0	0

**TABLE 2:** Demographic differences between survey respondents and community.

	Percentage of sample	Percentage of persons in the community
Trade or university qualification	72	49^a^
Hold AC/HCC	18	30^b^
First nations	< 1	7^c^

^a^ Australian Bureau of Statistics [27].

^b^ National Rural Health Alliance [28].

^c^ Australian Bureau of Statistics [29].

### 2.2 Interviews

Fifteen survey respondents consented to be contacted for an interview, four later withdrew their consent. Informed consent was obtained prior to the interviews. Eleven semi‐structured in‐depth interviews were conducted. According to the Australian Bureau of Statistics ‘Remoteness Areas’ [30], and the participants' residential postcode, seven resided within an ‘inner regional area’ and four resided in an ‘outer regional area’. All the participants were female and had one to two children under 5 years of age. They reported their children were generally well, apart from seasonal respiratory infections. Developmental delays were reported for one child, whereas a possible diagnosis of autism was flagged for another. No life‐threatening health concerns were noted. Five of the interviewees disclosed they were, or training to be a health or helping professional such as nursing, midwifery or a disability support worker.

A schema for the categories derived from the interviews is outlined in Figure 2. These categories are (1) Personal attributes and skills, (2) Seeking health information, (3) Quality health information, (4) Cautions and concerns and (5) Sequencing of searching.

#### 2.2.1 Personal Attributes and Skills

During the interviews, the participants identified that skills, in particular research skills learnt during university studies, were used when searching for children's health information. Some interviewees also reported relying on their instinct and intuition, because ‘if you've got your own concern (about your child), then you've got to trust your instinct' while (Participant 1).

#### 2.2.2 Seeking Health Information

Overwhelmingly the interview participants reported that ‘I do just google…’ when looking for health information. This was followed by selecting ‘just whatever google comes up with …’ (Participant 7). They stated they were more likely to search for information related to their child's illness rather searching health promotion topics. This appeared to be because health promoting behaviours were just ‘doing what's common sense’ (Participant 11), ‘and like I'm confident that we are already doing what we can’ (Participant 5).

The participants reported seeking health information from four sources, that is, the internet, GPs and specialised medical services, family and friends and other community‐based health services such as 13Health.

When using the internet, information was sought across three areas, that is, web sites, social media sites and blogs. The subject group reported they searched for ‘actual factual’ (Participant 7) or ‘proper information’ (Participant 10) from the internet, whereas social media sites were reported to be used for general information and for community support. For this group, blogs were reported to only be used when they were desperate for information, and these were reported to be the least reliable internet option.

When searching for ‘proper information’ participants sought out hospital or government web sites, well known organisation web sites such as the World Health Organisation as well as using the web sites their GP was known to use. A variety of social media sites over several platforms were also used for information. These included Facebook and Instagram pages. Sites were chosen by their relevancy to the current family situation such as ‘Babies due in January’ when expecting a baby in January. Preferred sites were described as not only being relevant to current needs, but also supportive with page users sharing information and experiences. One participant reported, ‘I do read a lot of those posts, and they are quite reassuring, because you do think it's not just me, you know, like it's not just it's not just my kid that does that weird thing’ (Participant 9) and another reported ‘it's just nice to see what other mums are experiencing at the same time, as what I am experiencing’ (Participant 4).

Blogs were viewed with great caution and used when ‘I was really desperate for answers’ (Participant 9). Scepticism about the quality of information on blogs was evident as ‘you don't necessarily know if they (the author/s of the blog) have a background of expertise or if they are doing this (product promotion) just for the money’ (Participant 10).

The GP plays a key role in the provision of health care for a family with young children [4]. The interviewees valued the GP as someone with expertise and knowledge who would do the best for their family because ‘Doctors have studied the profession…they have more of a knowledge base. And I believe that you know, if there's issues, I'm going to go to those doctors to get the right path’ (Participant 10). It was identified that if they have any concerns about the health of their child, they ‘definitely go straight to the GP’ (Participant 2), and that they ‘trust the GP more than google’ (Participant 1). In keeping with the experience of many Australian regional communities, access to the GP can be difficult with many participants experiencing waiting times and delays before having a consultation [4]. Some participants described access to a GP as an ‘absolute nightmare’ (Participant 4), with access times varying by locality. Although it was ‘just a lot harder in rural areas, with usually a two week wait, unless you call every morning’ (Participant 3), some participants living in larger towns reported they could get a doctor's appointment ‘if not that day, you might have to wait at least a day’ (Participant 8). Others reported having to call different doctors' practices within their area to get a timely appointment.

Communication with the GP was an important factor for the participants, as it allowed them to ‘check with my doctor on certain things’ (Participant 10), ‘and then ask them questions like “do you have information or like, do you have a recommended site we could look at more information for this …’ (Participant 8). Some participants stated they asked their GP where they could access further information, whereas others reported using internet pages recommended by their GP for health information or using the pages the GP had referred to during their consultation. None reported discussing their internet searches with the GP during a consultation.

Rapport with the GP was also valued with one participant noting, ‘we're just lucky we have such a good rapport with our doctors’ (Participant 7), while another shared, ‘I have a good relationship with my GP’ (Participant 10). An important component of the development of the rapport was being able to see ‘our normal doctor’ (Participant 8), as opposed to ‘another doctor in the practice’ (Participant 8) or having to visit an unfamiliar medical practice. This continuity was so important to one participant that she reported ‘we've been with the same GP centre now for 14 years’ (Participant 7).

Despite the recognised expertise of the GP, participants described questioning the GPs recommendations and double checking the information given to them. Participants expressed frustration at times with their GP visits such as when ‘nobody takes things seriously’ (Participant 9).

If participants were unable to access their usual GP in a timely manner, they reported that they would access the local hospital ED. This especially occurred after‐hours. Participants also reported accessing telehealth appointments, a ‘walk‐in’ doctors surgery, or the after‐hours home doctor service, if available in their town. One participant reported asking the GP for a referral to a specialist for a further opinion, and another made use of Cub Care services.^2^

The support and information supplied by family and friends was valued, especially support from friends with children a similar age to the participant's children. It was seen as ‘somewhere to check out ideas and information’ (Participant 5) and the support helped to ‘prevent unnecessary alarm’ (Participant 1). However, concerns were raised about the currency and validity of the information, with the information from family and friends described as ‘less reliable and out of date’ (Participant 9), and with the potential for the family to ‘downplay any concerns’ (Participant 1). But for one participant, family advice and information generated more worry as she found that ‘it weighs on my mind a lot’ (Participant 8).

The Queensland Government free 24‐h confidential health telephone service ‘13HEALTH’ had been utilised by all the interview participants. Some participants had valued the advice and information offered and felt it ‘was good just having someone there to give me a second opinion’ (Participant 11) and acknowledged ‘they do the best they can without seeing the patient’ (Participant 8). Other participants believed the information was unclear, and they were always referred for review to their GP. Participants also sought health information from their local pharmacist, Child Health services, Child's Personal Health Record booklet^3^ and medication information sheets. One participant reported her local playgroup invited a variety of health‐related guest speakers to share with the group, and it was ‘amazing, it was absolutely amazing that they do that’ (Participant 10).

#### 2.2.3 Quality Health Information

The interview participants sought quality appropriate health information for their children. They reported looking for information that was credible, relevant to their current needs and was immediately available.

Credibility of information was attributed to having an official source, that is a verified site. Government pages such as a ‘.gov.au’ page, health department, health agency and hospital sites were identified as being open to verification and therefore credible. Participants reported they checked the web address ‘…because that normally tells me whether that is a government website or not’ (Participant 10). Websites with industry backing such as a medical association were also identified as being open to verification and therefore the information was credible. Participants showed a preference for Australian web sites because ‘I guess you just want to lean towards something that you know is a bit more familiar. A bit more reliable …so it just puts my mind at ease a little more’ (Participant 2). Participants identified that it was important to know the ‘author, and if they've listed any associated qualifications like, you know, if they are a doctor, or if they have a degree of some sort’ (Participant 9). The author's experience and background were also important factors in identifying the credibility of the information.

Some participants looked for a consensus and consistency of information over three or four web sites as a means of verifying the information before adopting any recommendations, as one participant explained ‘I tended to like cross reference a couple of websites, … I wasn't just really going to just one source and as long as they are all fairly similar, then I treat it as fairly much what the consensus is’ (Participant 5). References to original sources and links to other pages of repute were also viewed favourably.

The presentation and appearance of a page was an important consideration when deciding the credibility of the site and the trustworthiness of information sourced online. The page needed to be ‘kind of neat and tidy, with easy‐to‐read text, like there's not information all over the place’ (Participant 3). The layout of the page contributed to the presentation with a preference for brief summaries, bullet points of salient issues, links to further information and guidance of when to see the doctor. The participants preferred information to be informative, succinct, well written and in an easy‐to‐read format. Ease of navigation across web pages and across the site was also used as an indicator of credible pages. Poorly written, untidy pages with multiple advertisements were viewed with suspicion. Pictures and photographs were advantageous to the participants because ‘there's actually so many websites that describe rashes without pictures and that is really annoying’ (Participant 5).

When searching for quality health information, the participants looked for information that was relevant to their current needs. Pages that ‘have a lot of information specifically targeted for little ones’ were the ‘one's that I probably take a bit more notice of’ (Participant 3) as opposed to general health information pages. Sites and pages that promoted specific information were also used such as ‘Boob to food’ when introducing solids and ‘Mumma matters’ for sleep and settling concerns with their baby. The participants described a sense of immediacy when looking for health information, that is ‘I just want the information right there, like the steps and straightforward information, rather than beating around the bush or finding all this information that you don't need. You kind of need the information fast’ (Participant 3).

#### 2.2.4 Cautions and Concerns About Health Information

Participants expressed an awareness about the volume of information that is available on the internet, with a participant stating, ‘there is so much of it out there, and it can be very hard for parents to wade through and work out what's actually reliable information and what they should be looking at’ (Participant 4). Care and caution were used when utilising online information with an unknown authorship because ‘you don't actually know who is giving that advice’ (Participant 10), and because ‘I've seen posts from Mum's groups online, and they recommend you put potatoes in the socks to leech out the toxins’ (Participant 11). Every participant expressed concern about drawing incorrect conclusions or deciding on the worst‐case scenario from the internet sourced information. As one participant expressed … ‘I guess like, Doctor Google who often comes to the conclusion and I think a lot of the times that it's like, you know, cancer or something awful’ (Participant 2). Consequently, ‘you can get a lot worried about stuff that you shouldn't be’ (Participant 1) because ‘you know as a mum you want to make sure you're doing the right thing and it can be a bit overwhelming sometimes, you know whether you have made the right decision or not’ (Participant 7). This concern generated caution as one participant stated ‘I always take it with a grain of salt. So, I don't read everything and believe it’ (Participant 7), and for one participant the concerns were such that ‘I stopped reading what is supposed to happen’ (Participant 11).

#### 2.2.5 Sequencing of Searching

The order of searching for health information was subject to the strength of the parental concern, that is, ‘it depends on how concerned I was’ (Participant 4), and ‘how unwell my child was’ (Participant 6). Most of the participants commenced their searching for health information on the internet. Subject to personal concerns and the severity of the illness, the participants then ‘checked in with friends’ (Participant 5), accessed primary health services such as 13 Health or the pharmacist and lastly sought review by the GP, unless ‘it was an emergency’ (Participant 10), when participants identified they would go straight to the hospital.

## 3 Discussion

To address the research questions of where and how parents with young children in regional and rural Southern Queensland search for health information, and to explore how they determine what is appropriate health information for their families, a short survey followed by in‐depth interviews was undertaken. The interview participants reported good health literacy, personal skills and professional knowledge that assisted them when looking for information for their children.

The participants reported searching the internet for health information in a similar manner to what has been reported in the literature, that is commencing the search with the google search engine [31, 32, 33], followed by choosing the first options listed by the search engine [34, 35]. Likewise, in keeping with the literature, the participants appraised the quality of the health information by checking the source of the information, the professional layout and appearance of the pages, the presence or absence of advertising, the readability of the internet page, the availability of external links, the credentials and qualifications of the authors, and the evidence of endorsements, such as health departments [34]. Although the participants used social media pages for support and shared experiences as has been described by others [36, 37, 38], they also indicated that webpages were utilised for ‘proper’ health information. This distinction in the usage of various internet platforms needs to be investigated further as it has potential implications for health services in the engagement of parents with health information and with the dissemination of child health information to targeted audiences. Interestingly, no one in this group searched the internet for health information related to wellness or health promoting activities, in part due to the perception that it is ‘common sense’. All health information searching was related to illness or sickness. This finding was unexpected as all participants had sound health literacy and many had a background in the health or helping industries. It had been surmised that if children of parents with low health literacy were less likely to experience preventative health care [15, 16], the reverse would appear likely, that is health literate parents would seek health promoting information for their children. Further exploration of this discrepancy is important to confirm these findings and to identify strategies that promote the uptake of child health information by parents.

The GPs expertise and knowledge were valued over online health information. This is congruent with the findings of Wainstein et al. [39], who reported 88% of Australians stated that they trust their doctor more than the internet. In contrast to the literature, where parents searched the internet to enable them to better describe their child's symptoms to the GP [32], and to formulate questions for the doctor [40], the participants in this study indicated that their internet searches prior to visiting the GP assisted with deciding when to access professional help. When communicating with parents, GPs and other health professionals could acknowledge that parents seek information from many sources including online sources, and that finding appropriate evidence‐based information can be challenging. These discussions may provide useful opportunities to explore where and how parents are searching for health information, and help parents to identify quality information.

Health information providers such as health services and government agencies may need to employ a variety of strategies to ensure the accessibility of the information. Easy‐to‐navigate web pages with information that is tailored and presented for those with low health literacy, in conjunction with well‐supported social media pages, are some steps that may contribute towards equitable health access.

### 3.1 Limitations

Despite extensive advertising, recruiting survey participants presented some challenges. As a result, the interview sample did not fully capture diversity in education, income and ethnicity. This aligns with existing research indicating that low socioeconomic and vulnerable populations are hard to reach and underrepresented [41]. These groups are more likely to visit EDs with their children [6] and face poorer long‐term health outcomes [2, 3]. However, this study highlights the importance of continued efforts to engage with these populations. By doing so, we can better understand and address the challenges of equitable health access, ensuring that all families receive the support and information they need for better health outcomes.

The findings of this study should be interpreted with caution due to the homogeneity of the sample, the small sample size, and the adequate health literacy levels of the participants. A larger sample size could have allowed for an analysis of the differences between those living in rural areas and those living in regional areas. Accessing parents from diverse backgrounds, including different states, would have given this study a broader understanding of the topic, and this would have been useful in furthering our understanding of how parents in rural and regional areas search for health information for their children.

## 4 Conclusion

The findings of this study indicate that parents of young children, who have good health literacy skills utilise a variety of strategies to find health information for their children that is credible and reliable. Health information sourced from hospital or government websites, or the GP was valued while health information sourced from family and friends was viewed with more caution. Web sites were used for ‘proper’ information while social media sites were accessed for support and connection. Parents used online health information to determine when to access their GP, and sought information for their child's illness or sickness, rather than health promoting information.

The insights offered by this research may inform the development of targeted Australian hospital and government websites to ensure all parents, with diverse skill levels, have easy access to appropriate evidence‐based children's health information. There may also be a role for GPs to discuss parents internet searches during a consultation, and to provide them with options for accessing further credible sources.

Further research is necessary to replicate these findings and to identify how parents with low health literacy skills seek and access appropriate children's health information, to enable the development of targeted effective health information.

## Author Contributions

**Catherine McCosker:** conceptualisation (lead), data curation (lead), formal analysis (lead), investigation (lead), methodology (equal), project administration (lead), writing – original draft preparation (lead), writing – review and editing (equal). **Gavin Beccaria:** supervision (equal), methodology (equal), validation (equal), writing – review and editing (equal). **Lisa Beccaria:** supervision (equal), methodology (equal), validation (equal), writing – review and editing (equal). **Tanya Machin:** supervision (equal), methodology (equal), validation (equal), writing – review and editing (equal).

## Conflicts of Interest

The authors declare no conflicts of interest.

## Ethics Statement

This project was approved by the University of Southern Queensland Human Research Ethics Committee (H22REA158).

## Data Availability

The data that support the findings of this study are available on request from the corresponding author. The data are not publicly available due to privacy or ethical restrictions.

## Endnotes

^1^
13Health is a Queensland Government confidential 24‐h health telephone service.

^2^
Cub care is a free online service that offers consultation with a paediatrician and is available for local residents though an agreement between Cub Care and the local Health Services.

^3^
The Personal Health Record is a booklet given to all parents in Queensland when their baby is born, which contains pertinent health information and provides a record of their child's growth and development, and immunisations.
